# Pars Plana Vitrectomy Combined with Anti-VEGF Injections as an Approach to Treat Proliferative Diabetic Retinopathy

**DOI:** 10.3390/jcm14155349

**Published:** 2025-07-29

**Authors:** Rafał Leszczyński, Wojciech Olszowski, Marcin Jaworski, Aleksandra Górska, Anna Lorenc, Irmina Jastrzębska-Miazga, Krzysztof Pawlicki

**Affiliations:** 1 Department of Ophthalmology, The Kornel Gibinski University Hospital Center, Medical University of Silesia, 40-514 Katowice, Polandmjaworski@uck.katowice.pl (M.J.);; 2 Department of Medical Biophysics, Medical University of Silesia, 40-752 Katowice, Poland; kpawlicki@uck.katowice.pl

**Keywords:** vitrectomy, anti-VEGF, sub-ILM haemorrhage, proliferative diabetic retinopathy

## Abstract

This study aimed to evaluate the impact of preoperative anti-VEGF injections on pars plana vitrectomy (PPV) outcomes in patients with proliferative diabetic retinopathy (PDR). **Material and methods:** We analysed 232 eyes with proliferative diabetic vitreoretinopathy treated with posterior vitrectomy. There were 112 women and 120 men. The patients were divided into two groups of 116 eyes each. In 116 eyes (study group), an anti-VEGF injection was administered 3 to 5 days before vitrectomy. The control eyes were not injected with anti-VEGF due to systemic contraindications to anti-VEGF treatment or lack of patient consent. All participants underwent pars plana vitrectomy with silicone oil injection. The oil was removed within 2–3 months after PPV. **Results:** At 2 years of observation, after removal of silicone oil, visual acuity (VA) was 0.24 ± 0.27 logMAR in the study and 0.37 ± 0.45 logMAR in the control group (*p* = 0.003). Intraocular pressure was 16.84 ± 6.25 mmHg in the study group and 17.78 ± 6.22 mmHg in the control group (*p* = 0.04). The mean duration of surgery was 47.62 ± 9.87 and 50.05 ± 9.41 min in the study and control groups, respectively (*p* = 0.02). The size of intraoperative haemorrhage was 0.97 ± 0.86 dd in the study group and 1.51 ± 1.22 dd in the control group (*p* = 0.003). The frequency of surgery-induced retinal breaks was 0.34 ± 0.56 in the study group and 0.56 ± 0.76 in the control group (*p* = 0.003). The recurrence rate of retinal detachment was 0.05 ± 0.22 in the study group and 0.1 ± 0.31 in the control group (*p* = 0.15). **Conclusions:** Preoperative anti-VEGF therapy shortens the duration of surgery, reduces complications, and improves long-term outcomes in terms of visual acuity and maintenance of normal eye function.

## 1. Introduction

Diabetes is the biggest epidemic of our time; 370 million adults aged 20–79 worldwide are affected, which is 1 in 10 people of this age. This number is expected to rise to 643 million by 2030 and 783 million by 2045. Proliferative diabetic retinopathy, a sight-threatening complication of diabetes mellitus (DM), affects approximately 6.87–7.04% of patients diagnosed with diabetes [1,2].

Diabetes causes generalised metabolic disorders, resulting in micro- and macroangiopathies and neuropathies that cause multi-organ complications affecting all organ systems. The most serious ophthalmic complications are proliferative diabetic retinopathy, macular oedema, and neovascular glaucoma. Significant advances in diagnostics, the use of artificial intelligence (AI) for screening, and new treatment methods increase the number of patients diagnosed at early stages and improve treatment outcomes [3,4,5,6].

Diabetic retinopathy can be classified into two types, i.e., non-proliferative diabetic retinopathy (NPDR) and proliferative diabetic retinopathy (PDR) with diabetic macular oedema (DME). Anti-vascular endothelial growth factor (anti-VEGF) drugs have become a vital treatment for diabetic retinopathy alongside laser therapy. However, almost 30–40% of patients with DR do not respond to anti-VEGF therapy, which necessitates the development of some other more effective treatments [6,7,8,9,10].

Untreated diabetes leads to the development of proliferative retinopathy, whose progression depends on the nature of diabetes and coexisting risk factors. The Wisconsin Epidemiologic Study of Diabetic Retinopathy revealed a 14-year progression rate of 86% for retinopathy, a 17% rate of retinopathy regression, and a 37% progression to proliferative retinopathy. The incidence of macular oedema was 26%. The risk factors for progression to proliferative retinopathy were more severe baseline retinopathy, male sex, higher glycosylated haemoglobin, and hypertension at baseline. These data suggest that reducing hyperglycemia and hypertension may result in decreased progression to proliferative disease [11].

The pathology underlying the development of proliferative diabetic retinopathy is retinal ischemia, which induces the production of vascular endothelial growth factors (VEGF), responsible for the proliferation of new pathological vessels along the vitreoretinal boundary/interface. Activated inflammatory mediators and other growth factors contribute to cell proliferation and extracellular matrix accumulation, resulting in the formation of preretinal fibrovascular membranes (FVM). Traction induced by fibrovascular membranes can cause tractional retinal detachments (TRD) and recurrent vitreous haemorrhages (RVH) [12,13,14].

### Therapy

Since the publication of the results of the Early Treatment of Diabetic Retinopathy Study (ETDRS), laser photocoagulation has been the standard and primary treatment for diabetic retinopathy, contributing to the delay of progression and reduction in complications [8,15].

Intravitreal anti-VEGF injections have become an important tool for preventing progression and complications of neovascularisation in both the anterior and posterior segments. On the other hand, some studies indicate that anti-VEGF therapy in eyes with PDR may aggravate fibrosis and shrinkage of the fibrovascular membranes, leading to complications such as tears, haemorrhages, or detachments [13,14,16].

The most common surgical treatment is pars plana vitrectomy (PPV). In 2021, Schreur et al. [17] presented long-term outcomes in patients who had undergone vitrectomy for proliferative diabetic retinopathy. The analysis included 217 eyes, with a follow-up period of 10 years. In most patients, functional visual acuity (VA) was achieved or maintained in at least one eye. After 10 years, approximately one quarter of all patients underwent re-vitrectomy, and more than half required vitrectomy of the fellow eye [17].

Mc Cullough et al. [18] analysed 406 studies involving 3839 eyes after vitrectomy due to diabetic retinopathy. The overall failure of retinal reattachment after one surgery was 5.9%, and the mean final visual acuity (VA) was 0.94 logMAR. Patients with higher preoperative VA achieved better postoperative vision (0.66 logMAR) but worse final vision. The results of this meta-analysis suggest that PPV is an effective strategy for attaining retinal reattachment in patients with tractional retinal detachment (TRD). The analysis of visual acuity may indicate that early surgical intervention should be considered and discussed with patients. To limit the development of retinal oedema and haemorrhagic complications, several authors recommend the use of anti-VEGF injections as pre-vitrectomy therapy [12,13,14].

Yue Xu [19] evaluated the concentrations of VEGF and fibrosis-related factors in the vitreous fluid of patients with PDR who had received preoperative intravitreal anti-VEGF injections (IVI) at various time points before pars plana vitrectomy. The results suggest that PPV should be performed within 5 days after IVI administration.

This study presents the significance of anti-VEGF pretreatment for PPV outcomes in patients with advanced diabetic retinopathy.

## 2. Materials and Methods

The paper presents an analysis of 232 eyes (232 patients) with proliferative diabetic retinopathy who underwent posterior vitrectomy with silicone oil endotamponade. All patients had pseudophakia in the anterior segment; the intraocular lens had been properly implanted. The posterior segment exhibited haemorrhage of varying severity and proliferative diabetic retinopathy without macular involvement, as confirmed with ocular ultrasound.

Preoperative examinations revealed the presence of rubeosis iridis, retinal proliferation, and tractions caused by existing proliferative changes. The analysis of the presented preoperative changes in both groups is shown in Table 1.

The study included 125 women and 107 men who had been operated on at the vitreoretinal surgery department between 2010 and 2021. The surgeries were performed by three experienced surgeons at one centre in two vitreoretinal departments.

The patients were divided into study and control groups, with 116 eyes each. The mean age of the patients in the study group was 57.18 ± 14.33 years.

The study group received an intravitreal anti-VEGF injection (Ranibizumab 0.5 mg/0.05 mL) 3 to 5 days before surgery. The eyes of the control patients with proliferative diabetic retinopathy were not injected with anti-VEGF due to drug hypersensitivity, a history of stroke, myocardial infarction, or generalised cardiovascular disease. The study and control groups did not differ regarding the number of patients, intraocular haemorrhages, rubeosis, vitreoretinal traction, and preretinal proliferation.

The study group showed worse baseline visual acuity than the control group (*p* = 0.0001). Preoperative ocular ultrasound confirmed the presence of haemorrhage, retinal proliferation, and traction in all patients, without macular involvement. The ultrasound findings were confirmed by intraoperative observations.

All patients underwent PPV with silicone oil injection. The oil was removed within 2–3 months after PPV.

### 2.1. Inclusion Criteria

The analysis included only patients who attended the follow-up examination two years after surgery. All patients had proliferative retinopathy and pseudophakia, with the intraocular lens correctly positioned within the lens capsule.

Patients qualified for the study had macula-on retinal status, with no evidence of epimacular proliferation on ultrasound examination.

All patients had impaired fundus visualisation due to intraocular haemorrhage of varying severity. The study encompassed individuals with iris rubeosis, vitreoretinal traction, vitreous haemorrhage, and other proliferative changes. The preoperative characteristics of both groups are presented in Table 1.

### 2.2. Exclusion Criteria

Patients with proliferative diabetic retinopathy and intact lens, PDR and history of iritis, patients with PDR and diabetic keratopathy, patients with PDR and an implant in the ciliary sulcus, patients with PDR and a subluxated intraocular lens, aphakic patients, and patients with neovascular angle-closure glaucoma, and those with previously diagnosed high-grade optic neuropathy were excluded.

### 2.3. Surgery

All procedures were performed under local anaesthesia, using a lidocaine and bupivacaine mixture. Anaesthesia was supported with remifentanil (Ultiva) administered via an infusion pump in amounts depending on the patient’s discomfort.

**Figure 1:**
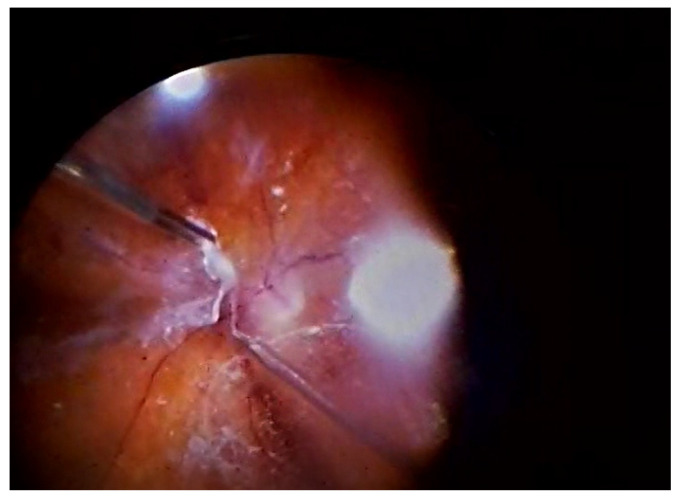
Bimanual sutureless 23G pars plana vitrectomy.

Bimanual sutureless 23G pars plana vitrectomy was performed in each patient. A 25G chandelier endoillumination system was also placed, allowing for a bimanual surgical procedure (Figure 1). PPV was started with central and peripheral vitrectomy. In the next stage, an attempt was made to detach the posterior vitreous body, controlling and releasing the traction so as not to cause iatrogenic retinal tears. After diathermy of the vascularized lesions, the preretinal proliferations were removed. After staining with Brilliant Peel dye, fragmentation and dissection of the preretinal membranes and ILM in the macular area were performed using a bilateral technique. In the next stage, fluid/air exchange was performed, and decalin was administered. Following proliferation removal, laser photocoagulation was applied to the periphery, retinal lesions, and along blood vessels. All iatrogenic tears that formed during the removal of the proliferation (1500–2000 coagulates, E-170 mW) were sealed during laser therapy. Vessel arcades were only lasered in cases of significant changes that posed a potential threat to the macula. The procedure was completed with silicone oil application; all patients were administered peribulbar depo-medrol. The oil was removed within 3–4 months after PPV.

### 2.4. Statistical Analysis

Statistical calculations were performed using Statistica version 10 PL (Statsoft, Tulsa, OK, USA). The Shapiro–Wilk test was used to determine whether the data followed a normal distribution, and the Levene test was used to assess the homogeneity of variance. The Mann–Whitney test, a non-parametric equivalent of Student’s *t*-test, was used to compare the study and control groups. The significance level was set at *p* < 0.05.

## 3. Results

### Course of the Procedure

In patients with PDR, pars plana vitrectomy is associated with a risk of bleeding. The estimated haemorrhage size ranged from one to four disc diameters (dd) in both groups. The mean intraoperative bleeding was 0.97 ± 0.86 dd in the study and 1.51 ± 1.22 dd in the control group (*p* = 0.003), and was significantly higher in the control group. Postoperative hyphema was found on the first day after surgery in 0.12 ± 0.33 of the study eyes and 0.28 ± 0.45 of the control eyes (*p* = 0.003), resolving spontaneously within 14 days. Anterior chamber paracentesis at the slit lamp was required in two eyes of the control group.

Visual acuity at 2-year follow-up after silicone oil removal was 0.24 ± 0.27 logMAR in anti-VEGF-treated eyes and 0.37 ± 0.45 logMAR in the control group (*p* = 0.0003). The intraocular pressure was 16.84 ± 6.25 mmHg in the study group and 18.78 ± 6.22 mmHg in the control group (*p* = 0.04). The mean surgery duration was 47.62 ± 9.87 and 50.05 ± 9.41 min in the study and control group, respectively (*p* = 0.0002).

Follow-up period hypotonia, associated with vascular membrane detachment, was found in 0.08 ± 0.27 of the study eyes and 0.1 ± 0.31 of the control eyes (*p* = 0.4).

At 2 years of surgery, intraocular pressure (IOP) was 16.84 ± 6.25 mmHg in the study and 17.78 ± 6.22 mmHg in the control group (*p* = 0.04). IOP fluctuations were observed in 0.26 ± 0.44 of the study eyes and in 0.47 ± 0.5 of the control group (*p* = 0.0006), necessitating transscleral cyclophotocoagulation (TSCPC) in 0.25 ± 0.49 of the study eyes and 0.3 ± 0.52 of the control eyes (*p* = 0.36). Two study eyes in the study group and three control eyes underwent a repeat TSCPC.

The patients used anti-glaucoma medications to maintain intraocular pressure within the target range. The number of anti-glaucoma drugs was 0.48 ± 1.11 in the study group and 0.67 ± 1.22 in the control group (*p* = 0.2). Table 2 presents the post-surgery comparison of the study and control groups.

The administration of anti-VEGF injections had no significant effect on the number of anti-glaucoma medications, TSCPC procedures, or the number of patients with hypotony (*p* > 0.05).

## 4. Discussion

Since the ETDRS, laser therapy has been the standard treatment for diabetic retinopathy, significantly slowing the progression of diabetic lesions [15,20]. However, the method has drawbacks that limit its use. Koca and Kilic [20] concluded that both conventional and pattern laser photocoagulation performed in diabetic retinopathy patients caused an increase in central macular thickness (CMT) and thinning of the retinal nerve fibre layer (RNFL) over an extended observation period. These changes were more noticeable in the conventional laser group compared to the pattern scan laser group.

The use of anti-VEGF alone in the treatment of proliferative diabetic retinopathy is a matter of controversy. On the one hand, it limits angiogenic activity, but on the other hand, it may increase the proliferative activity of the connective tissue and shrinkage of fibrovascular membranes. In advanced PDR, anti-VEGF may induce the so-called crunch effect and result in retinal tear and haemorrhage [21].

Administering anti-VEGF injections before vitrectomy can be combined with post-vitrectomy laser coagulation. Photocoagulation performed after proliferative tissue removal allows for the reduction in complications and increases the scope of the intervention to include areas affected by haemorrhage and proliferation [20,21]. We did not observe any complications after the administration of anti-VEGF and after PPV, during which thorough laser coagulation of the retina was performed.

Proliferative retinopathy in young patients poses a significant challenge. Chen et al. [22] investigated the impact of intravitreal ranibizumab injections on complications following vitrectomy in young patients with proliferative diabetic retinopathy. Anti-VEGF injections were administered 3 to 5 days before surgery. The authors found that the total duration of surgery was shorter in the group treated with ranibizumab compared to the control patients; however, they did not observe any significant differences in the prevalence of late recurrent vitreous haemorrhage (VH), neovascular glaucoma (NVG), recurrent retinal detachment, or final visual acuity.

In our study, we found fewer intraoperative haemorrhages and haemorrhages within the first 24 h after surgery, fewer retinal tears, and better postoperative visual acuity in eyes with preoperative anti-VEGF treatment. These better outcomes might be attributed to the fact that intraoperative bleeding significantly reduces visibility within the surgical field, necessitating an increase in blood pressure and blood removal. Similarly to Chen et al. [22], we observed prolonged surgical times in the control group (no pretreatment with intravitreal anti-VEGF), and we did not find any differences in the prevalence of secondary retinal detachments.

Li et al. [23] evaluated the efficacy and safety of intravitreal injection with Ranibizumab (IVR) before vitrectomy for severe PDR associated with vitreous haemorrhage. IVR-treated eyes exhibited less bleeding and fewer retinal tears during vitrectomy, and no recurrence of tractional retinal detachment. The authors concluded that IVR administered 1 to 3 days prior to surgery could significantly reduce VEGF content in the aqueous humour, thereby effectively optimising intraoperative conditions. Our analysis also revealed a reduction in surgery time, a decrease in the number and area of haemorrhages, and fewer retinal tears during surgery. However, anti-VEGF treatment did not protect against the development of TRD.

Pérez-Argandoña et al. [24] searched the Epistemonikos database for studies on pre-vitrectomy administration of bevacizumab into the vitreous. They found that such pretreatment reduced the rate of vitreous haemorrhage in the early and late postoperative periods. They also emphasised that the effect of bevacizumab on visual acuity was not entirely clear and added that anti-VEGF injection probably shortened the duration of surgery and might reduce the development of iatrogenic retinal tears. Our follow-ups showed better visual acuity in the group after preoperative anti-VEGF administration; this difference was statistically significant after 2 years.

Smith and Steel [25] reviewed the effects of pre- or intra-PPV injection of an anti-VEGF (bevacizumab) on the incidence of complications and the condition of the eyeball. The authors believe that the preoperative or intraoperative use of bevacizumab reduces the incidence of early postoperative vitreous cavity haemorrhage (POVCH).

Our study group achieved better visual acuity than the control group, and there were fewer cases of hyphema. However, we did not find a significant difference in the prevalence of recurrent retinal detachments. This may be because, in pseudophakic eyes, the vitreous clearance is typically more precise, which may reduce the likelihood of recurrent vitreous haemorrhages.

Anti-VEGF injections administered prior to pars plana vitrectomy (PPV) in patients with proliferative diabetic retinopathy (PDR) do not completely prevent intraoperative haemorrhages or other surgical complications. Nevertheless, this approach appears to reduce both the number and extent of complications and significantly shortens the duration of the procedure. These benefits contribute to improved anatomical and functional outcomes compared to patients who do not receive adjuvant anti-VEGF therapy.

Administration of anti-VEGF injections prior to PPV does not significantly impact the incidence of hypotony or the number of secondary retinal detachments during the two-year observation period.

Further studies are required to elucidate the factors affecting surgical outcomes and complication rates in patients undergoing treatment for proliferative vitreoretinopathy. These factors include pseudophakia, pharmacological treatments, haematological parameters, and timing of surgical intervention.

## Figures and Tables

**Table 1:** Pre-PPV group characteristics.

Groups	Study Parameters	Age—Years	Visual Acuity logMAR (Operated Eyes)	Intraocular Pressure (Operated Eye)	Visual Acuity (Non-Operated Eye)	Intraocular Pressure (Non-Operated Eye)	Rubeosis	Proliferations	Tractions
Study Group (anti-VEGF)	Mean and Standard deviation	57.18 ± 14.33	0.14 ± 0.14	17.37 ± 3.39	0.40 ± 0.31	16.49 ± 4.09	0.14 ± 0.35	0.84 ± 0.37	0.26 ± 0.44
	Range	23.0–90.00	0.01–0.60	7.00–28.0	0.01–1.04	5.00–40.0	0.0–1.00	0.0–1.00	0.00–1.00
Control Group (no anti-VEGF)	Mean and Standard deviation	62.35 ± 12.98	0.10 ± 0.20	17.33 ± 4.06	0.30 ± 0.34	16.34 ± 2.98	0.08 ± 0.27	0.83 ± 0.38	0.23 ± 0.42
	Range	23.0–90.0	0.00–1.00	5.00–37.00	0.00–1.81	6.00–25.00	0.00–1.00	0.00–1.00	0.00–1.00
	*p*	0.0064	0.0001	0.89	0.0001	0.97	0.15	0.86	0.65

**Table 2:** Characteristics of patients after the procedure during the last examination.

Group	Study Parameters	Intraoperative Haemorrhage	Retinl Breaks	Visual Acuity (Operated Eye)	IOP—(Operated Eyes)	Visual Acuity (Non-Operated Eyes	IOP—(Non-Operated Eyes)	Anterior Chamber Hyphema	Retinal Detachment	Ocular Hypotony	Glaucoma	Surgery Duration	Repeat Surgery	Number of Anti-Glaucoma Drugs	TSCPC
Study group (anti-VEGF)	Mean and SD	0.97 ± 0.86	0.34 ± 0.56	0.24 ± 0.27	16.84 ± 6.25	0.26 ± 0.35	17.65 ± 4.42	0.12 ± 0.33	0.05 ± 0.22	0.08 ± 0.27	0.26 ± 0.44	47.62 ± 9.87	0.23 ± 0.46	0.48 ± 1.11	0.25 ± 0.49
	Range	0.00–4.00	0.00–2.00	0.00–1.00	4.00–40.00	0.00–3.00	5.00–30.00	0.00–1.00	0.00–1.00	0.00–1.00	0.00–1.00	30.00–95.00	0.00–2.00	0.00–4.00	0.00–2.00
Contro group (no anti- VEGF)	Mean and SD	1.51 ± 1.22	0.56 ± 0.76	0.37 ± 0.45	17.78 ± 6.22	0.31 ± 0.24	18.79 ± 3.88	0.28 ± 0.45	0.10 ± 0.31	0.10 ± 0.31	0.47 ± 0.5	50.05 ± 9.41	0.12 ± 0.33	0.67 ± 1.22	0.30 ± 0.52
	Range	0.00–4.00	0.00–3.00	0.01–4.00	4.00–33.00	0.00–1.00	11.00–26.00	0.00–1.00	0.00–1.00	0.00–1.00	0.00–1.00	1.00–70.00	0.00–1.00	0.00–4.00	0.00–2.00
	*p*	0.003	0.03	0.003	0.04	0.03	0.04	0.003	0.147	0.49	0.0006	0.02	0.04	0.19	0.35

IOP—Intraocular pressure.

## Data Availability

Are available in section “MDPI Research Data Policies” at https://www.mdpi.com/ethics.

## Abbreviations

PDR	Proliferative diabetic retinopathy
DM	Diabetes mellitus
RD	Diabetic retinopathy
AI	Artificial intelligence
DME	Diabetic macular oedema
NPDR	Nonproliferative diabetic retinopathy
VEGF	Vascular endothelial growth factor
NCVH	Non-clearing vitreous haemorrhage
CMT	Central macular thickness
FVM	Fibrovascular membranes
VH	Vitreous haemorrhage
RNFL	Retinal nerve fibre layer
NVG	Neovascular glaucoma
TRD	Tractional retinal detachment
POVCH	Postoperative vitreous cavity haemorrhage
TSCPC	Transscleral Cyclophotocoagulation
